# Rapid, label-free surface plasmon resonance discrimination between *Bothrops* and *Crotalus* venoms using clinical antivenom as the capture reagent

**DOI:** 10.1371/journal.pntd.0013501

**Published:** 2026-01-06

**Authors:** Ely S. F. Borges, Jomar S. Vasconcelos, Arthur A. Melo, Fernanda C. C. L. Loureiro, Karla P. O. Luna, Antonio M. N. Lima

**Affiliations:** 1 Graduate Program in Biochemistry and Molecular Biology, Federal University of Rio Grande do Norte, Natal, RN, Brazil; 2 Graduate Program in Cellular and Molecular Biology, Federal University of Paraíba, João Pessoa, PB, Brazil; 3 Electrical and Electronics Department, Federal Institute of Maranhão, São Luís, MA, Brazil; 4 Biosensing Laboratory, Federal University of Campina Grande, Campina Grande, PB, Brazil; 5 Department of Electrical Engineering, Federal University of Campina Grande, Campina Grande, PB, Brazil; 6 Department of Biology, State University of Paraíba, Campina Grande, PB, Brazil; Fundação de Medicina Tropical Doutor Heitor Vieira Dourado: Fundacao de Medicina Tropical Doutor Heitor Vieira Dourado, BRAZIL

## Abstract

Snake envenoming is recognized as a global health problem, affecting thousands of people every year. One of the main challenges in addressing this issue is the correct identification and treatment of these envenomations, particularly in locations where people do not have easy access to hospitals. In Brazil, the genus *Bothrops* is responsible for the majority of envenomations, followed by *Crotalus*. This study reports a simple methodology for detecting crude venom from snakes of the genus *Bothrops*, through interaction with their corresponding antibodies, using a high-sensitivity optical biosensor. The protocol consists of adding antibodies (present in the commercial antivenom) to the sensor surface, followed by the addition of *Crotalus* venom (nonspecific), and then *Bothrops* venom (specific), resulting in changes in the refractive index, to evaluate cross reactions between them. Different concentrations of raw venom from snakes of the genus *Bothrops* and *Crotalus* were tested, starting with a concentration of 6.784 μgmL^−1^ and progressing until reaching the minimum detectable concentration. The binding capacity of venom to antivenom was investigated at two concentrations of antivenom: 5 μgmL^−1^ and 50 μgmL^−1^. Both antivenom and snake venoms were solubilized in phosphate-buffered saline (PBS). The results show an accurate detection of the antigen of interest (*Bothrops* venom), tested at different concentrations. The biosensor was able to detect venom up to a concentration of 0.848 μgmL^−1^. In addition, no interference from nonspecific binding between the *Bothrops* antivenom and *Crotalus* venom was detected. The detection of specific venom (*Bothrops*) occurred in a satisfactory time (up to 14 minutes). The results provide evidence that the biosensor and the methodology employed can be considered a diagnostic model under development, which can help health workers to better identify and treat envenomated people.

## Author summary

This study was conducted to address the critical need for rapid and accurate snakebite diagnosis by developing a specific optical biosensor methodology capable of distinguishing genera *Bothrops* from *Crotalus* venom, by using thus clinical antivenom facilitating timely and effective antivenom treatment. We developed a label-free Surface Plasmon Resonance (SPR) biosensor using clinical *Bothrops* antivenom (BAV) to detect *Bothrops* venom, finding that the device could rapidly and specifically identify the venom at concentrations as low as 0.848 $\mu {\text{gmL}}^{-1}$ with no cross-reactivity to *Crotalus* venom. These findings indicate that the developed SPR biosensor serves as a rapid, highly sensitive, and reusable diagnostic tool capable of accurately distinguishing *Bothrops* from *Crotalus* envenomation using BAV, thereby offering a promising solution for improving clinical decision-making and treatment effectiveness in resource-limited settings.

## Introduction

Snake envenoming is a serious health problem that can lead to death [1], since the toxins present in the venom of these animals can cause damage of varying magnitudes to physiological systems [2]. According to the World Health Organization (WHO) [1], 4.5 to 5.4 million people are bitten by snakes annually. Among these, 1.8 to 2.7 million develop clinical diseases and 81,000 to 138,000 die from complications. However, due to underreporting, the true extent and impacts of snakebites are still unknown [3]. In Brazil, the families Viperidae (genera *Bothrops* and *Bothrocophias*, *Crotalus* and *Lachesis*) and Elapidae (genera *Micrurus* and *Leptomicrurus*) comprise the venomous snakes of relevance to public health, and snakes of the genus *Bothrops* are responsible for the largest number of accidents [4].

Snake venoms are composed of up to 90% proteins; some families of these proteins have hundreds of isoforms [5]. Snake envenomation can cause severe paralysis (potentially compromising respiration), hemorrhagic disorders, irreversible kidney damage, and necrosis of the tissues surrounding the bite site. These complications may ultimately result in permanent disability or limb amputation [1]. The clinical manifestations of snake envenomation vary according to the chemical composition of each venom: Envenomations by snakes of the Elapidae family usually induce neurotoxic, cytotoxic, and cardiotoxic manifestations, while envenomations by snakes of the Viperidae family usually cause myotoxicity and hemotoxicity [2].

High-quality antivenoms (which follow appropriate production standards and undergo rigorous evaluation before being tested in patients) represent the most efficient therapeutic approach to prevent most of the impacts resulting from snake envenomation and are officially recognized as essential medicines by the WHO [1] in combination with support treatment [6]. The success of antivenom therapy depends on the correct and early diagnosis of the snake involved in the envenomation, therefore, ensuring access to rapid diagnostics can promote better results [7]. The clinical decision regarding which specific antivenom to administer is based on a comprehensive patient history—including clinical manifestations, circumstances of the bite, initial care provided, description of the snake, clinical signs, and medical background—along with local epidemiology and laboratory tests such as clotting time (CT), activated partial thromboplastin time (aPTT), D-dimer and/or fibrin degradation products (FDPs), complete blood count, creatine kinase (CK) levels, electrolytes, urea, blood urea nitrogen/creatinine, and urinalysis [6]. Although these methods are essential for clinical monitoring, they are limited by infrastructure, operational costs, supply availability, and the need for trained personnel—critical challenges in rural areas where most snakebite incidents occur [2]. Consequently, there is an urgent need for diagnostic tests that can overcome these barriers and provide rapid, reliable results.

Differentiating envenomation caused by snakes of the genera *Bothrops* and *Crotalus* remains a significant challenge in clinical practice. While local tissue damage and systemic coagulation disturbances are characteristic of *Bothrops* envenomation, and systemic myotoxicity, renal injury, and neurotoxicity predominate in *Crotalus* envenomation [8], clinical overlap, delayed symptom onset, and lagging laboratory markers can occur. These factors impede early and accurate identification, which is essential for the prompt selection of appropriate antivenom [9]. In view of this, it is crucial to develop rapid, highly reliable and specific tests to detect the type of envenomation to provide better treatment effectiveness [7,10,11].

The usage of biosensors has contributed to great advances in the health area, thus, there is a growing need to develop simple, sensitive and economical diagnostic tools, such as biosensors, to effectively detect diseases [12]. A progressive interest in the application of sensor technologies for snake detection has been reported [13]. Researchers have been working on developing biosensors capable of detecting snake venom. However, the application of these tests in the field remains uncertain due to obstacles such as long analysis time, non-reuse of chips, high-cost equipment and reagents, meticulous preparation of the test, and the need for improvements in detection [14,15].

Biosensors are analytical devices that make biological signals measurable by changes generated by interactions between biological components, converting them into an electrical signal [12]. A conventional biosensor consists of the following elements: analyte, bioreceptor, transducer, electronic components, and a system for interpreting the results [16,17].

Given the urgent need to develop highly reliable and sensitive tests capable of accurately and quickly determining the occurrence of snakebite envenomation and the type of snake that caused the accident, this research proposed to develop a simple and rapid methodology using a highly sensitive optical SPR biosensor for detecting venom of the snakes of the genus *Bothrops* by using *Bothrops erythromelas* venom.

## Materials and methods

Samples of crude venom from snakes of the species *Bothrops erythromelas* were obtained from adult specimens from the Museu Vivo Répteis da Caatinga, located in the state of Paraíba, Brazil. The venom was kept at a temperature of -20°C until use. Venom samples from snakes of the species *Crotalus durissus terrificus* were provided by the Instituto Vital Brazil, located in the state of Rio de Janeiro, Brazil. The *Bothrops* antivenom (BAV) was provided by the Health Department of the State of Pernambuco and kept refrigerated at 4°C. The anti-Bothrops serum used is produced from a combination of venoms from five species of snakes of the *Bothrops* genus (*B. jararaca*, *B. alternatus*, *B. jararacussu*, *B. moojeni* and *B. neuwiedi*) and its composition consists of F(ab’)2 fractions of heterologous, specific and purified immunoglobulins [18]. Both the antivenom serum and the snake venoms were solubilized in phosphate-buffered saline (PBS), pH 7.2, immediately before the experiments.

As for the study concentrations of *Bothrops* and *Crotalus* venoms, a series of preliminary assays were carried out to establish the optimal concentration range capable of generating measurable and reproducible SPR responses without signal saturation. The maximum venom concentration adopted (6.784 $\mu g\;mL^{-1}$) corresponds to the lowest concentration previously used by our group in calibration tests involving similar biosensing setups, which consistently produced a strong and stable interaction curve. To determine the minimum detectable concentration, serial dilutions of the crude venom were prepared in phosphate-buffered saline (PBS, pH 7.2) at ratios of 1:15,000, 1:30,000, and 1:60,000, the preparation of serial dilutions was supported by studies that employed similar approaches in biosensors [19,20]. Each dilution was tested sequentially under identical flow and timing conditions until the lowest concentration still producing a detectable resonance shift was identified as 0.848 $\mu g\;mL^{-1}$—a value consistent with the range of serum concentrations reported for human envenomation [21]. This procedure defined the dynamic range of detection for the proposed assay.

Regarding the BAV, two working concentrations were established to evaluate the influence of surface antibody density on binding efficiency. The stock BAV (purified $F(ab^{\prime }{)}_{2}$ immunoglobulin fraction) was diluted to 5 $\mu g\;mL^{-1}$ (1:1000) and 50 $\mu g\;mL^{-1}$ (1:100) in PBS. These values were selected based on previous optimization experiments performed by our group to determine the coating concentration that ensures complete IgG immobilization without steric hindrance effects, allowing effective antigen recognition on the gold surface. Both concentrations were tested under the same experimental conditions to assess differences in signal amplitude and specificity.

**Fig 1:**
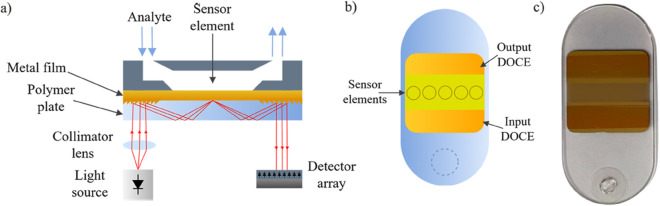
Schematic (not in scale) of SPR instrument set-up (a) with an exchangeable polymer chip with optical grating couplers, input DOCE and output DOCE (b), and the photograph of used chip (c).

For the experiments, we employ a Surface Plasmon Resonance (SPR) instrument that operates in angular interrogation mode (AIM). The instrument comprises a red laser light source, lenses for optical beam coupling, an image detector, and electronic components [22]. Fig 1 depicts the schematic diagram of the SPR sensor. The SPR phenomenon refers to the coupling between electromagnetic and surface plasmon waves, on the interface of two different media with different dielectric constants (normally, a metal film and a dielectric). When the light excitation condition is fixed, the SPR technique allows precise measurement of changes in the: 1) refractive index; 2) thickness of the medium adjacent to the metal film; and 3) adsorption layer on the metal surface [23,24]. In AIM, a significant loss in reflected light at a specific angle for a fixed light source wavelength is observed. As the venom material diffuses out of the serum solution, the SPR instrument indicates the shift in the angular position of the intensity minimum. Here, it is represented as a variation of the effective refractive index (RIU), displayed as a so-called SPR-sensorgram.

**Fig 2:**
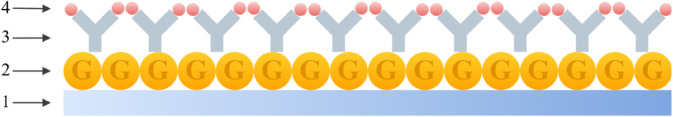
Representation of the arrangement of the layers. 1. polymer substrate with a 2. thin 50 nm gold layer (G, in yellow); 3. antibodies (Y, in gray); 4. Snake venom (spheres in red). The solutions were transported to the biochip by a peristaltic pump. Flow is essential due to the need to measure multiple analytes in a single assay. The flow rate varies between 5 and 10 μLmin^−1^, values directly related to the analyte, to optimize the adsorption and desorption process of the analyte on the gold film.

Fig 2 shows a representation of the arrangement of the layers formed, which follows the following order of addition: first the antivenom serum (containing antibodies), followed by the *Crotalus* snake venom and, lastly, the *Bothrops* snake venom (to assess the presence or absence of cross-reaction between the anti-Bothrops serum and the *Crotalus* venom simultaneously with the test to assess the specificity between the anti-Bothrops serum and the *Bothrops* venom).

Initially, the gold surface of the biochip was washed with deionized water while the biochip was outside the sensor, followed by a second wash with deionized water after coupling the biochip to the sensor to avoid any possible air contamination, as well as to have a reading that started at zero, since deionized water was used as a reference. From the moment the water flow entered, the signal began to be measured and recorded. The flow was paused for a few seconds so that the input channel could be manually transferred to the next solution. The graphs were generated when the interactions on the sensor surface occurred.

**Fig 3:**
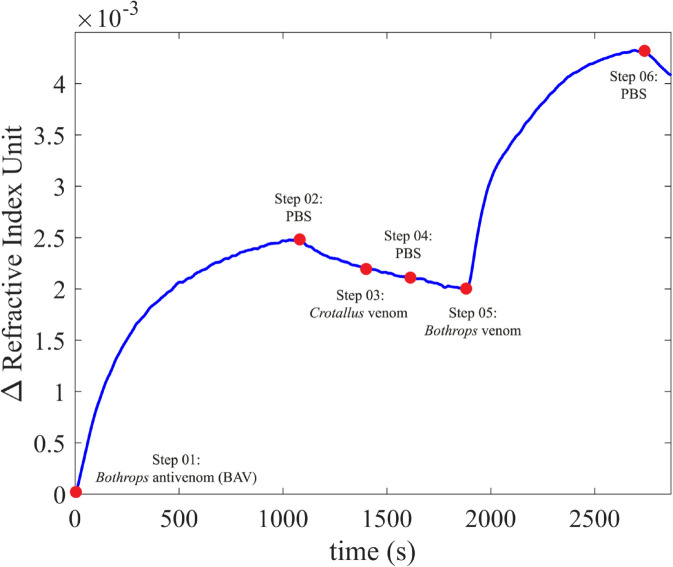
Illustrative graph demonstrating the test stages that make up the results. The blue markers on the graph indicate the time point at which the type of fluid in the pump reservoir was changed.

Fig 3 illustrates how the results are presented, following the steps described below. Step 1: the BAV diluted in PBS was admitted; the physical adsorption of the antibodies on the gold was evidenced by the increase in the line on the graph, and when the line became constant (indicating total coverage of the film) the flow was paused. Step 2: the inlet channel was transferred to the PBS solution, so that the weakly bound molecules were removed (desorption process), an event indicated by the decay of the line on the graph; the flow was paused. Step 3: the inlet channel was transferred to the *Crotalus* venom solution diluted in PBS; after the line on the graph became constant, the flow was paused. Step 4: PBS was admitted again, and, after the line on the graph remained constant, the flow was paused. Step 5: the inlet channel was transferred to the *Bothrops* venom solution diluted in PBS; when the line on the graph became constant, the flow was stopped. Step 6: the inlet channel was transferred to the PBS solution so that the molecules (present in the venom) weakly bound to the previous layer were removed.

At the end of each experiment, a cleaning cycle was performed to remove the substances adsorbed on the sensor surface. This consisted of flowing deionized water over the surface, followed by enzymatic detergent (Extran) diluted in deionized water, and then deionized water again. This washing procedure did not cause any damage to the gold film. After the surface cleaning step, a new experiment could be performed using the same chip. All tests were performed in triplicate.

The experiments were performed at the Biosensors Laboratory of the Department of Electrical Engineering (DEE) - Center for Electrical and Computer Engineering (CEEI), at the Federal University of Campina Grande (UFCG), located in the state of Paraíba, Brazil.

To analyze kinetic interactions, we apply an approach based on the one-to-one Langmuir binding model, which assumes homogeneous binding sites and no mass transport limitations. Considering the interaction between the sensor chip surface (G) and the injected analyte (A) following the one-to-one Langmuir model for complex formation (AG) in a flow-cell-based SPR system, continuous flow maintains A constant. Therefore, during analyte injection phase, A is equal to *C*_*A*_, and during the buffer (PBS) injection phase, A is equal to zero. The sensor response varies with time according to the ordinary differential equation [25,26]:

$$\frac{dR}{dt}=k_{on}C_{A}(R_{max}-R)-k_{off}R,\;\text{with }C_{A}\neq 0\text{ for }t_{0}\leq t\leq t_{1},$$ (1)

$$\frac{dR}{dt}=-k_{off}R\;\text{for }t\geq t_{1},$$ (2)

where, *R* is the observed response and *R*_*max*_ corresponds to the maximal response that would be observed if an infinite concentration of A was injected. *k*_*on*_ represents the association constant and *k*_*off*_ the dissociation constant.

By analytically solving the ordinary equation under the assumption of homogenous binding sites. If no mobile analyte has been initially bound to the gold surface, the time course of association is described by an exponential equation [27]:

$$R(t)=R_{eq}(1-e^{-k_{obs}t}),$$ (3)

$$R_{eq}=\frac{k_{on}C_{A}R_{max}}{k_{on}C_{A}+k_{off}},\;\text{and}\;k_{obs}=k_{on}C_{A}+k_{off}.$$ (4)

If the free analyte is removed from the buffer ($t\geq t_{1}$), the complex AG dissociates exponentially with time as expressed in the equation [27]:

$$R(t)=R_{0}e^{-k_{off}(t-t_{1})}.$$ (5)

in which, *R*_0_ represents the sensor response at the start of dissociation. In this model, the observed binding rate constant *k*_*obs*_ in the association phase is always higher than the dissociation rate constant *k*_*off*_.

In our analysis, the model parameters $\theta =[R_{eq},k_{obs}]$ for association phase and $\theta =[R_{0},k_{off}]$ for dissociation phase and were optimized by minimizing the sum of squared residuals (SSR) between the experimental data *R*(*t*) and the model prediction $\hat{R}(t;\theta )$ as follows:

$$\theta^{*}=\underset{\theta \in {\mathbb{R}}^{2}}{\text{argmin}}\sum_{i=1}^{N}[R(t_{i})-\hat{R}(t_{i};\theta ){]}^{2},$$ (6)

where, *N* is the number of data points. The constrained nonlinear least squares problem was solved using the trust-region reflective least squares algorithm [28,29]. The algorithm adopted a termination tolerance on the function value of 10^−6^ with a termination tolerance on θ of 10^−6^. We consider as constraints the lower and upper bounds given by $0<R_{eq}<\infty$ and $0<k_{obs}<\infty$. As initial guesses, we considered θ=[max(R(t)),0.1] with $t_{0}\leq t\leq t_{1}$ for association phase and $\theta =[R(t=1),10^{-3}]$ with $t\geq t_{1}$ for dissociation phase.

## Results

**Fig 4:**
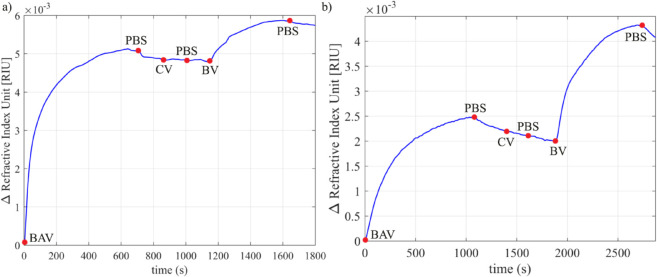
Graphs of the moving average of the refractive indices of PBS, BAV, *Crotalus* Venom (CV) and *Bothrops* Venom (PB) as a function of time. a) BAV 1:100, CV 1:7,500, PV 1:7,500; b) BAV 1:1,000, CV 1:7,500, PV 1:7,500. The blue markers on the graph indicate the time point at which the type of fluid in the pump reservoir was changed.

**Fig 5:**
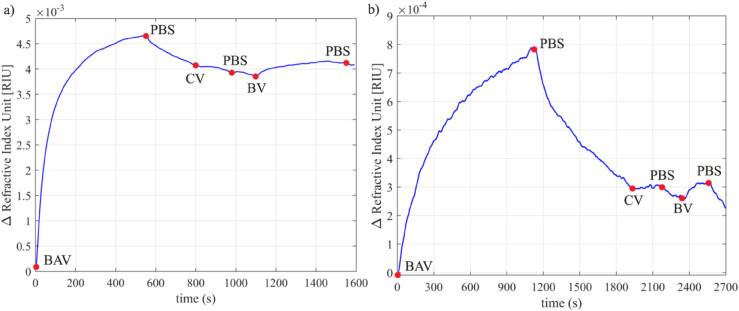
Graphs of the moving average of the refractive indices of PBS, BAV, *Crotalus* Venom (CV) and *Bothrops* Venom (PB) as a function of time. a) BAV 1:100, CV 1:60,000, PV 1:60,000; b) BAV 1:1,000, CV 1:60,000, PV 1:60,000. The blue markers on the graph indicate the time point at which the type of fluid in the pump reservoir was changed.

The interaction between antibodies and antigens was evaluated at different concentrations of venom and antivenom, by the change in the refractive index for each substance (Figs 4 and 5). In the experiments, the binding of BAV to the sensor surface and the binding capacity of venom to the IgG layer were investigated at two different concentrations of BAV. The results of the interaction between antibodies and the corresponding (*Bothropic*) and non-corresponding (*Crotalus*) venom are shown in Figs 4 and 5.

It is possible to observe the change in the sensor response as the antibodies and antigens interact, as shown in the curves shown in Figs 4 and 5. Each curve corresponds to the adsorption/desorption of a specific substance, showing the deposition of layers on the sensor surface, confirming the conjugation between bioreceptor and analyte. The sensor signal tends to return to the baseline when the flow containing venom is interrupted and exchanged for PBS, due to the dissociation process. Although at a concentration of 1:60,000, *Bothrops* venom dissociates more easily from antibodies due to its low concentration, the sensor detected the adsorption of this venom.

The intensity of the sensor response to venom was proportional to the concentrations of venom and IgG (BAV) used. The highest venom concentration (6.784 μgmL^−1^) generated a higher response when using the lowest IgG concentration (5 μgmL^−1^), on the other hand, when using the highest IgG concentration (50 μgmL^−1^), the refractive index of the venom at high concentration was lower. When we tested the lowest venom concentration (0.848 μgmL^−1^) together with the lowest IgG concentration, there was a higher refractive index of the venom; at the high IgG concentration the refractive index of the venom was lower. From Figs 3A and 4A, it is possible to observe that as the venom protein concentration increased from 0.848 μgmL^−1^ (venoms in a ratio of 1:60,000) to 6.784 μgmL^−1^ (venoms in a ratio of 1:7,500), the refractive index increased by 3.91, compared to immunoglobulins at a concentration of 5 μgmL^−1^. Similarly, Figs 4B and 5B show that as the venom protein concentration increased from 0.848 μgmL^−1^ to 6.784 μgmL^−1^, the refractive index increased by 0.75, compared to immunoglobulins at a concentration of 50 μgmL^−1^. Regarding the biosensor response, the complete experiment time for the tests varied between 25 and 40 min, depending on BAV concentration. It is important to note that the biosensing detection time should be measured from the moment the venom admission to the flow cell. The detection time varied from 5 to 14 min (cf. Figs 4 and 5).

**Fig 6:**
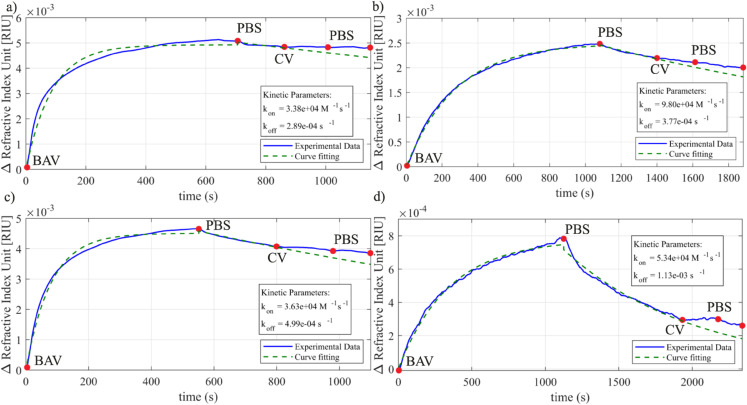
Kinetics constant estimation for different concentration of BAV. a) BAV 1:100, CV 1:7,500, PV 1:7,500; b) BAV 1:1,000, CV 1:7,500, PV 1:7,500; c) BAV 1:100, CV 1:60,000, PV 1:60,000; d) BAV 1:1,000, CV 1:60,000, PV 1:60,000.

Figs 4 and 5 also reveal the absence of a variation in the refractive index of *Crotalus* venom on *Bothrops* antibodies, thus confirming the absence of cross-interaction. We analyzed the adsorption of IgG (BAV) by implementing curve fitting to estimate the association and dissociation constants. The experimental data (represented by the blue line) is plotted alongside the estimated data (shown as a dashed green line) for the experiments in Fig 6.

To estimate the kinetics parameters, we considered the association time as the time comprised in the interval between the red dot BAV and the first red dot PBS. The dissociation time corresponds to the interval between the first red dot PBS and CV injection. After estimating the parameters, we apply the values in Eqs. (4) and (6), covering the dissociation interval from the first PBS injection to the end of the second PBS injection cycle. According to the estimated curve (dashed green line), we would have a higher quantity of BAV removed from the sensor surface by injecting only PBS instead of CV. However, the second PBS cycle removed all possible molecules in the CV solution, approximating the sensor response to the estimated dissociation curve and showing the specificity of BAV.

To confirm the selectivity between *Bothrops* antibodies and *Bothrops* venom, *Bothrops* venom was promptly added after the passage of the *Crotalus* venom (both venoms at the same concentration). With the presence of *Bothrops* venom, the occurrence of interaction between the BAV and *Bothrops* venom was observed, thus, the previous passage of the fluid containing the *Crotalus* venom did not affect the interaction between the corresponding antibodies and venom. Which means that, even when submitted first to the system, the *Crotalus* venom is not capable to interact or modify the link between *Bothrops* venom and its specific antivenom. Which could happen as venoms share similar epitopes [30] and can elicit cross-reactions [31]. This data shows that the SPR sensor allied to the simple methodology developed, is capable to go forward and be used to discriminate between these two venoms at point of care institutions.

## Discussion

In this article, we report a simple methodology for discriminate between *Bothrops* and *Crotalus* venoms using a surface plasmon resonance biosensor, based on the evaluation of antigen-antibody interaction. To more accurately reproduce the real situation, we used antivenom as a bioreceptor, since it is the most common and accessible form of obtaining antibodies in health centers. We also used crude snake venom, considering that this is the form in which venom can be found in body fluids available for evaluation, such as blood and urine.

The study involved two representative species of the family *Crotalidae*: *B. erythromelas* and *C. d. terrificus*. *B. erythromelas* was selected because it accounts for the highest incidence of snakebite accidents in the Northeast region of Brazil and belongs to the genus of greatest medical relevance in the country [32]. *C. d. terrificus*, in turn, was included as a reference species due to its wide distribution across Brazil and because it is part of the venom pool used for the production of the anticrotalic antivenom [33].

Although the Brazilian antivenom used for treating *Bothrops* envenomation—and employed in this study—is produced from the venoms of five species [18], *B. erythromelas* is not included in its immunizing pool. Nevertheless, antivenoms are capable of neutralizing homologous and heterologous venoms from phylogenetically related species, despite their broad biochemical and immunological diversity [34]. Considering that treatment is based on administering a genus-specific antivenom, detecting antigen–antibody interactions at the genus level is sufficient to guide clinical management.

In line with these observations, the ability of seven polyspecific antivenoms, produced in six Latin American countries, to neutralize venoms from different *Bothrops* species was evaluated by [34]. They observed a pattern of broad cross-neutralization, demonstrating the capacity of these antivenoms to neutralize both homologous and heterologous *Bothrops* venoms. Another study [32] showed that the Brazilian antibothropic antivenom was able to neutralize most of the toxins present in *B. erythromelas* venom—particularly those with hemorrhagic activity—confirming a significant interaction between the antivenom and the venom of this species. Conversely, a study [35], which tested the ability of the Brazilian antibothropic antivenom to neutralize venoms from different *Bothrops* species, reported intermediate antibody titers against *B. erythromelas* venom, with Phospholipase A2 (PLA2) activity neutralization below 10%.

It is important to note that antibody–antigen binding by affinity and antigen neutralization by antibodies, although related, are not always equivalent processes; an antibody may recognize and bind to a toxin without necessarily blocking its effector function [36]. The lack of neutralization of certain *B. erythromelas* toxins by the antibothropic antivenom does not pose major implications for this study, since our interest lies in understanding the extent to which antibodies bind to structurally similar antigens. In this context, assessing molecular-level recognition is entirely sufficient to meet the objectives of the study.

Two groups of tests (Figs 4 and 5) were performed; in each group a specific concentration of antibodies was used and the venom concentration varied. It was observed that the refractive index of the antibody layer was different for each test in which the same concentration of BAV was used. This difference in response, even using the same concentration of BAV, can be attributed to the packaging of antibodies [37], which, due to immobilization by physical adsorption, can occur in different ways, since in this type of adsorption the antibodies spontaneously bind to the surface of the sensor, causing a disordered orientation [38–40].

Figs 4 and 5 reveal that the presence of high venom concentrations results in a greater shift in the resonance angle compared to tests in which low venom concentrations were applied (which resulted in a smaller shift in the resonance angle). Thus, the existence of a direct relationship between the concentration of the substance and the intensity of the response is demonstrated [19,41].

Two different concentrations of IgG were used to evaluate the intensity of venom binding; thus, a relationship was observed between the concentration of antibodies and the amplitude of the venom response. When the antibody solution was less concentrated, the venom at a high concentration elicited a more intense response. In contrast, in the experiments in which the antibody solution was more concentrated, the venom at a high concentration resulted in a less pronounced response. High surface coating densities have been shown to reduce antibody binding capacity due to spatial constraints, as antibodies obstruct each other’s access to binding sites [42]. Thus, at a lower concentration, the binding capacity between the IgG domain and the antigen is increased.

An important attribute observed was the reusability of the chip used: after each test was completed, the surface cleaning procedure to remove the adsorbed substances proved to be efficient, since the signal returned completely to its baseline, established by passing a flow of deionized water. Thus, subsequent measurements were not affected by the later measurements. This data is significant because underlines the importance of reusability of this type of system on a point of care institution, even more if it is supported by public financial incentive.

Regarding snake venom composition, it is important to emphasize that variability occurs across multiple taxonomic levels, from families to individuals of the same species [43]. This diversity arises both from genetic mutations, genetic drift, and natural selection—which shape venom components according to the functional demands of each snake group [30], and from other factors such as prey type, geographic location, age, and habitat quality [43]. However, some toxin families, due to the homology of venom glands, are shared among the three snake families that possess front-fanged dentition [30]. Toxins within the same family may exhibit specific antigenic features resulting from divergent evolutionary processes, while still sharing common structural and functional characteristics [31]. This structural conservation may, in turn, promote cross-reactivity between antivenoms and venoms of non-corresponding genera [44,45]. Thus, the balance between conserved and divergent antigenic features in toxins determines whether an antivenom will cross-react with heterologous venoms and inhibit their biological activity [46].

Within the family *Crotalidae*, snake venoms are characterized by high complexity and considerable heterogeneity in toxicity [47]. In the genus *Bothrops*, snake venom metalloproteinases (SVMPs) constitute the predominant toxin family, followed by PLA2, snake venom serine proteases (SVSPs), and L-amino acid oxidases (LAAOs). In contrast, *Crotalus* venom contains PLA2 as its major component, followed by SVMPs, SVSPs, and defensins (crotamine) [48].

As a result of the differential occurrence of toxins between these genera—both in proportion and in toxin families—biosensor-based studies have proven effective in distinguishing them. By developing impedimetric immunosensors for snakebite diagnosis, assessed the potential for cross-reactivity between antibothropic antibodies and venoms from different genera and detected no significant variations indicative of immunocomplex formation between the antibody and the heterologous venom [19,20]. Both studies are consistent with our findings, which show no resonance shift suggestive of cross-reactive interactions between BAV and *Crotalus* venom (Figs 4 and 5), confirming the selectivity of BAV for *Bothrops* venom and the accuracy of the biosensor in discriminating the specific interaction.

The concentrations of *Viperidae* venoms (to which the genera *Bothrops* and *Crotalus* belong) in human adult plasma range approximately from 0.005 to 0.9 $\mu g\;mL^{-1}$, based on ELISA assays [21]. A major limitation noted in serum venom concentration data is the pronounced dispersion of reported values. Such discrepancies may be related to multiple factors, including variation in the absorption rate of venom components due to their broad range of molecular weights [49] and the variable amount of venom injected [50]. Additionally, because ELISA-based assays rely on multiple antibodies to detect multiple antigens, they may not accurately reflect the true characteristics or variability of individual toxins within a biological system [21]. Thus, developing more precise methods and studies to quantify venom in humans is essential for generating more robust and reliable data.

The results of this study demonstrated that the biosensor was capable of detecting *Bothrops* venom at concentrations as low as 0.848 $\mu g\;mL^{-1}$. Although this value is close to the lower limit of physiopathological concentrations of clinical interest, it remains within the range of serum venom concentrations in humans reported by [21]. This initial work aimed to validate the feasibility of detecting snake venom from the genus of greatest medical relevance in Brazil using an SPR-based biosensor. Our findings provide a necessary baseline for subsequent optimizations aimed at enhancing sensitivity, including strategies such as oriented antibody immobilization, refinement of the biotechnological protocol, and analyses using human biological fluids. In this way, our study not only seeks to address a limitation in the clinical management of snakebite envenomation but also offers a path toward rationalizing and improving antivenom use, enabling more precise and safer administration of these therapeutic agents.

In the present study, a genus-specific *Bothrops* antivenom was employed, which provided the selectivity required to demonstrate the feasibility of the biosensor. This approach is consistent with the aim of the work, namely to demonstrate the viability of bioreceptor immobilization and the ability of the SPR system to selectively detect *Bothrops* venom through its interaction with antibodies developed against the same genus. Because this strategy depends on molecular specificity to ensure signal selectivity, the use of a polyvalent antivenom—produced from mixtures of venoms from different genera [51]—could compromise detection accuracy, as such antivenoms exhibit a heterogeneous antibody profile, representing an important limitation. However, it is important to emphasize that polyvalent antivenoms are indeed used in clinical practice when identification of the snake responsible for the envenomation is difficult [51]. Thus, our study not only seeks to address a limitation in clinical management but also offers a pathway to improve antivenom use.

A SPR sensor was developed for detecting crude venom from the Indian snake Naja naja and a polyvalent antivenom produced against four snake species found in India: Naja naja, Bungarus caeruleus, Daboia russelii, and Echis carinatus [52]. As in our study, all measurements were carried out in phosphate-buffered saline, and the influence of other blood components was not evaluated. After testing different antibody concentrations and incubation times, all sensors were constructed using 2 mg/mL of antibody incubated for 8 hours, and the venom diluted in PBS was exposed to the sensor for 12 minutes.

The SPR biosensor used by the authors detected venom at concentrations between 0.1 $\mu g\;mL^{-1}$ and 1.0 $\mu g\;mL^{-1}$, whereas the SPR biosensor used in our research showed superior performance, achieving a detection limit of 0.848 $\mu g\;mL^{-1}$. This difference may be related to the authors’ use of a polyvalent antivenom as the biorecognition element. Another relevant limitation of the study [52], resulting from the use of a polyvalent antivenom, is the inability to determine the genus of the snake responsible for the envenomation; the response is restricted to the presence of toxins, since the signals may arise from interactions with venoms of any of the genera covered by the polyvalent serum profile.

A label-free impedimetric immunosensor to detect different concentrations of venoms from snakes of the genera *Bothrops*, *Crotalus*, and *Micrurus*, diluted in PBS, was proposed by [19], achieving a detection limit of 0.1 $\mu g\;mL^{-1}$ using affinity-purified antibodies. In contrast, our study obtained a detection limit of 0.848 $\mu g\;mL^{-1}$ by directly employing commercial antivenom [19]. They used affinity-purified antibodies—a labor-intensive process that selectively isolates only the immunoglobulins specific to the target venom—the biosensor used in our study proved sensitive to the interaction between the venom and the commercial antivenom (composed of $F(ab^{\prime }{)}_{2}$ fragments) distributed throughout the Brazilian healthcare system. This demonstrates that the biosensor can be used directly for the biorecognition of *Bothrops* venom, making the technology more accessible and cost-effective, as it relies on a reagent widely available in healthcare networks.

Moreover, we employed crude *Bothrops* venom, whose use provides greater biological fidelity because it corresponds to the material actually inoculated during envenomation and eliminates costly steps of toxin fractionation and purification. The use of crude venom maintained good specificity toward the antibothropic serum, as evidenced by the absence of response to the heterologous *Crotalus* venom and by the concentration-dependent signal, confirming that the observed binding is physiologically relevant. Thus, the biosensor demonstrated consistent analytical performance, reinforcing the feasibility of a simpler, more accessible approach that remains aligned with the real context of snakebite envenomation.

Recent advances in venom biosensing include electrochemical platforms based on nanostructured materials and modified electrodes. A multiplexed electrochemical immunosensing system using graphene/gold nanoparticle–modified screen-printed electrodes for the simultaneous detection of snake and scorpion venoms was introduced by [53], achieving high sensitivity and multi-analyte capability, but requiring redox mediators, electrode surface modification, and dedicated electrochemical instrumentation. Similarly, an ultrasensitive electrochemical immunosensor based on Sm–Co doped antimony–tungstate nanostructures was reported by [54], in which signal amplification relied on complex nanomaterial functionalization, linker-assisted antibody immobilization, and redox-dependent transduction mechanisms, reaching detection limits in the sub-*ng mL*^−1^ range, though at the cost of increased fabrication complexity and reduced portability. In a different context, a screen-printed gold electrodes functionalized with anti-Bungarus candidus IgG for venom quantification in plasma from experimentally envenomed rats was emplyed by [55], using impedance and voltammetric readouts based on charge-transfer resistance modulation. Although these approaches demonstrate excellent analytical performance and robustness in controlled laboratory or animal model conditions, they inherently depend on electrochemical architectures, surface microfabrication steps, and redox-active systems.

In contrast, the present study adopts a fully optical and label-free surface plasmon resonance (SPR) strategy, enabling real-time monitoring of antigen–antibody interactions without electrical mediation, nanomaterial doping, or chemical signal amplification. By employing crude *Bothrops* venom and a commercial antibothropic serum as the recognition layer, the proposed SPR platform prioritizes experimental simplicity, reproducibility, and closer alignment with real diagnostic scenarios. The absence of electrode modification and redox probes significantly reduces preparation time and sources of variability, while the non-destructive and non-invasive optical readout allows repeated measurements with minimal surface degradation. Therefore, rather than competing solely on detection limits, this work contributes a complementary design philosophy centered on operational practicality, reduced complexity, and direct adaptability to portable SPR-based diagnostic systems for rapid identification of *Bothrops* envenomation in clinical and field settings.

## Conclusion

This study demonstrates a rapid, label-free SPR biosensor that distinguishes Bothrops from Crotalus venoms using clinical antivenom directly immobilized as the capture agent. The sensor sensitively and specifically detected Bothrops venom at clinically relevant concentrations (down to 0.848 $\mu g\;mL^{-1}$) without cross-reactivity to Crotalus, and its response correlated with venom and antibody levels. The device remained selective with crude venom samples and was reusable after regeneration, supporting its practical application at the point of care. Compared to more complex electrochemical and nanomaterial-based platforms, this SPR approach offers simplicity, label-free optical detection, and uses reagents readily available in healthcare settings. While the current sensitivity meets clinical needs, further optimization of bioreceptor orientation could improve performance. Although genus-specific antivenom limits broader application, this method represents a significant advance in accessible, rapid snakebite diagnostics. Overall, these findings highlight the potential of SPR biosensing for timely, accurate snakebite diagnosis and targeted antivenom treatment, particularly in resource-limited regions. Further validation using human samples and field studies will be essential for full clinical implementation in endemic areas.

## Supporting information

S1 File Experimental dataset. Ensure that you have a working installation of MATLAB from MathWorks. Before running any of the MATLAB scripts (‘plot_figure_6a_4a.m’, ‘plot_figure_6b_4b.m’, ‘plot_figure_6c_5a.m’, and ‘plot_figure_6d_5b.m’) to generate the figure plots (Figs 4, 5, and 6), make sure all required files are unzipped into the same folder. Then, set MATLAB’s current directory to this folder before executing the scripts to avoid file path issues. This will help ensure that all dependencies are accessible and the scripts run without errors.
(ZIP)
